# Effects of vegetation restoration and environmental factors on understory vascular plants in a typical karst ecosystem in southern China

**DOI:** 10.1038/s41598-020-68785-7

**Published:** 2020-07-21

**Authors:** Zhiyang Ou, Shilong Pang, Qinfei He, Yuhua Peng, Xiaorong Huang, Wenhui Shen

**Affiliations:** Institute of Forest Ecology, Guangxi Forestry Research Institute, No. 23, Yongwu Road, Nanning, 530002 People’s Republic of China

**Keywords:** Community ecology, Community ecology

## Abstract

Understory vegetation is an important component in most forest ecosystems. It is very important for soil and water conservation in karst region, study on understory will provide valuable information for understanding the interaction mechanism between understory flora and karst environment. Thirty-two plots were sampled in three vegetation types along with a restoration gradient (shrubland, forest–shrub transition, and mixed-species broadleaf forest) in typical karst mountains in Southwest Guangxi, China. Overstory trees, understory vascular plants, soil nutrients, and topographic factors were recorded in each 400-m^2^ plot. Multivariate statistics were used, including the multi-response permutation procedure (MRPP), indicator species analysis, and canonical correlation analysis (CCA). MRPP showed understory species composition significantly differed among the three vegetation types, with the greatest difference between the shrubland and the mixed forest. Twenty-one understory species were identified as significant indicator species, with 13 species being identified as indicators of the shrubland, two of forest–shrub transition, and six of the mixed forest. Light-demanding herbaceous seed plants were common in shrubland, while shade-tolerant calcicole assembled under the mixed forest. Forward selection of CCA ordination revealed that understory plant distribution was most strongly influenced by elevation, followed by soil pH, the concentration of total potassium and exchangeable calcium, slope aspect, slope degree, and the concentration of available potassium. The result reveals that vegetation types affect understory species composition by modifying understory environments. Elevation affects the spatial distribution of vegetation and soil factors, and then the understory plants. Meanwhile, soil Ca content also plays a key role in the understory species distribution. Understory diversity increased with increasing canopy structure complexity from shrubland to mixed-species forest. Thus, it is necessary to take measures to promote natural vegetation restoration and to protect the mixed forests in degraded karst areas.

## Introduction

Understory flora represents the important component of biodiversity and plays a key role in maintaining structure and ecosystem functions in forests^1–3^. Although understory vegetation contributes relatively little to the total forest vegetation biomass, it contributes a great proportion of floristic diversity^2,4^. In some areas of the Hyrcanian zone, herbaceous plants constitute over 90% of the total plants with diverse life forms^5^. Understory flora also influences soil enzyme activity and soil fertility and improves material cycling and energy flow in forest ecosystems^6,7^. Understory flora provides a nursery, facilitates the colonization of target plants, improves the survival and growth of tree seedlings, changes the understory microhabitat, and affects forest hydrology^8–10^. Study on understory flora could help improve understanding community dynamics, plant responses to disturbance, and forest regeneration and helps guide conservation and management^11–17^.

The forest canopy is a major determinant in the forest ecosystem. It strongly influences the understory flora through heterogeneous microhabitat caused by species composition and structure^18–20^. Thus, understory species composition differs markedly among forests^21,22^. Previous research found forest canopy controls understory flora by modifying understory environments and resource availability by competing with understory plants for resources both above and below ground^23–25^. Giesbrecht et al. stated that overstory structure drives fine-scale spatial structure in the understory plant community by light transmission^26^. Studies have demonstrated a significant relationship between understory flora and soil-chemical gradient modified by canopy cover^27,28^. For instance, Bartels and Chen^29^ asserted that increasing overstory broad-leaved tree abundance increased the richness of herbaceous-layer plants via its impact on soil nutrients. In addition, topography partially influences microenvironment by controlling the spatial redistribution of light, temperature, water availability, and soil nutrients and, thereby, affects understory flora. In hilly regions, slope aspect exerts an especially strong influence on understory flora, with northeast-facing plots exhibiting higher values of species richness and Hill’s diversity than southwest-facing plots^30,31^. The spatial patterns of understory flora are also closely associated with slope aspect^32,33^. In comparison, other studies have shown that the richness and diversity of understory species vary with elevation, not with slope aspect^34,35^.

The karst landforms of China are mainly distributed in the southwestern region, where the ecological environment is fragile and exhibits a low resistance to interference. Intensive human activity, combined with the distinct geological background, is leading to the continuous degradation of forests to shrubland and grassland. In some karst areas, rocky desertification or seasonal rocky desertification has appeared, with large areas of bare bedrock or block accumulation. The natural vegetation is sparse in karst rocky desertification area, and the soil and water loss is quite serious. Therefore, the ecological security of this area has been severely affected, so ecosystem restoration and reconstruction of the vegetation is urgent. Many studies have focused on the spatial patterns of forest communities, and the vegetation dynamics and restoration in this region^36–38^. The current study was carried out in Pingguo County, southwest Guangxi, China. There is a large area of typical karst landforms, with an appropriate area of rocky desertification. After many years of ecological conservation, there has been a noticeable increase in forest coverage. During this period of ecological conservation, the economic value of ecological restoration and soil quality under different vegetation restoration models were studied^39,40^. Some studies demonstrated that the spatial patterns and diversity of woody plant species were significantly affected by soil and topographic factors^41,42^. Understroy is very important for soil and water conservation in karst region, but few studies have addressed the understory in this area. Whether the factors affecting arbor layer also impact the understory, and how the environmental factors systematically interpret the species diversity variation of understory? Knowledge about it in this area is still lacking. Therefore, we examined understory vascular plant communities under three types of overstory canopy (shrubland, forest–shrub transition, and mixed-species broadleaf forest) in a typical karst ecosystem and attempted to elucidate the effects of vegetation types and environmental factors (soil nutrients and topography) on the understory. Our specific objectives were to: (1) detect whether and how vegetation type controls the composition and distribution of understory flora and (2) assess the relative importance of soil properties and topographic features in regulating the understory flora. We expect that our findings will provide valuable information for understanding the interaction mechanism between understory flora and karst environment in the karst areas.

## Materials and methods

### Study area

The study was carried out in the karst mountains of Pingguo County (23° 12′–23° 51′ N, 107° 21′–107° 57′ E), located in southwestern Guangxi, China. The landforms of Pingguo are mainly composed of karst, where the vegetation very easily degrades, leading to rocky desertification. In 2014, areas without obvious rocky desertification in Pingguo County only accounted for 18.47% of the total karst area^43^. The climate of the area is of the south subtropical monsoon. Annual average temperature ranges from 19 to 21.5 °C, and annual mean precipitation ranges from 1,200 to 1,500 mm and mainly occurs from May to September^41^. The soil has been identified as brown and black limestone soil. Due to a long history of human interference, the local vegetation has deteriorated across large areas. The prevailing vegetation types are shrub–grassland, shrubland, open forest, and secondary forest. The shrubland is dominated by *Vitex* *negundo*, *Cipadessa* *cinerascens*, and *Alchornea* *trewioides*. The remained secondary forests are patchily distributed near villages, and the tree layer is mostly dominated by *Excentrodendron* *hsienmu*, with common species including *Sapium* *rotundifolium*, *Ficus* *concinna*, *Choerospondias* *axillaris*, and *Walsura* *robusta*.

### Sampling

In 2012, we established 32 plots of 20 × 20 m, of which 9 plots were established in shrubland, 11 plots were in forest–shrub transition forest (forest–shrub), and 12 plots were in mixed-species broadleaf secondary forest (mixed-forest). Field investigations were carried out in May and June 2012. Woody plants with DBH (diameter at breast height at 1.3 m, measured to 0.1 cm accuracy) ≥ 1.0 cm and height ≥ 1.5 m were defined as stand tree. In each plot, the DBHs and heights of stand trees were recorded. The details of canopy characteristics are found in Table 1. Understory plants with heights < 1.5 m were censused inside five 2 × 2 m quadrats, with one in the centre and four at the corners of the plot. In each quadrat, data were collected regarding the number of vascular plants, height, and coverage by species. Data from each quadrat were pooled for the plot-level analyses. Topographic factors were recorded, including the ratio of rock bareness (Rock), elevation (Elv), slope degree (Degree), slope aspect (Aspect), and slope position (Position).

**Table 1:** Mean values of environmental factors and characteristics of overstory and understory flora of three vegetation types (means ± SD).

Items	Shrubland (n = 9)	Forest-shrub transition (n = 11)	Mixed-species broadleaf forest (n = 12)
Soil pH	6.63 ± 0.08^b^	6.66 ± 0.05^b^	7.01 ± 0.11^a^
Soil organic matter/g kg^-1^	105.41 ± 5.00^b^	168.23 ± 7.16^a^	170.55 ± 20.49^a^
Total nitrogen/g kg^-1^	5.64 ± 0.32^b^	9.17 ± 0.37^a^	9.59 ± 0.82^a^
Total phosphorus/g kg^-1^	1.86 ± 0.06^a^	1.91 ± 0.14^a^	2.23 ± 0.22^a^
Total potassium/g kg^-1^	4.26 ± 0.59^b^	1.9 ± 0.22^b^	7.61 ± 1.47^a^
Available nitrogen/mg kg^-1^	345.89 ± 23.45^b^	577.97 ± 19.15^a^	668.83 ± 53.95^a^
Available phosphorus/mg kg^-1^	1.14 ± 0.16^b^	1.34 ± 0.08^ab^	2.45 ± 0.58^a^
Available potassium/mg kg^-1^	116.23 ± 6.25^a^	82.8 ± 3.82^b^	136 ± 14^a^
Exchangeable calcium/mg kg^-1^	3,029.48 ± 233.61^b^	3,447.8 ± 269.59^ab^	3,935.23 ± 315.18^a^
Ratio of rock bareness/%	50.88 ± 6.2^b^	75.34 ± 4.16^a^	86.36 ± 2.65^a^
Slope degree/°	36.67 ± 2.64^b^	46.82 ± 2.63^a^	40 ± 3.92^ab^
Elevation/m	319.11 ± 7.32^ab^	334.18 ± 3.45^a^	298.17 ± 15.64^b^
Understory number of individuals	7,317	4,914	7,033
Understory species richness	91	85	138
Understory evenness	0.555	0.784	0.682
Understory *α*-diversity index	2.502	3.481	3.362
Overstory species richness	91	111	119
Overstory evenness	0.782	0.792	0.819
Overstory *α*-diversity index	2.638	2.750	2.755
Overstory density/trees·ha^−1^	8,436.11 ± 2,890.86^a^	9,322.73 ± 4,471.01^a^	3,862.50 ± 1713.27^b^
Overstory height/m	3.65 ± 0.66^b^	4.09 ± 0.72^b^	5.52 ± 1.09^a^
Overstory DBH/cm	2.08 ± 0.29^b^	2.64 ± 0.47^b^	4.77 ± 1.13^a^

*α*-diversity index refers to the Shannon–Wiener index (*H*') at the site level.

Different letters indicated that the differences in each item within a row were significant at *P* < 0.05.

Because the soil is shallow in the karst mountains, soil samples were only collected at a depth of 0–15 cm at eight to ten random locations along an S-shaped transect in each plot. Samples were mixed to obtain approximately 1 kg per 20 × 20 m plot for subsequent chemical analysis. The soil samples were air-dried in the laboratory and sieved through a 0.25-mm mesh screen. The soil was tested at the Soil and Research Division of the Guangxi Forestry Research Institute, China, following the methods prescribed in Bao^44^: soil pH value was measured with a acidity meter; soil organic matter (SOM) was measured by using potassium dichromate oxidation-outer heating; total nitrogen (TN) and available nitrogen (AvN) were measured by using distillation method, total phosphorus (TP) was analysized by using NaOH melting-molybdate antimony anti color development-UV Spectrophotometry method; available phosphorus (AvP) was measured by using 0.5 mol L^−1^ NaHCO extraction-molybdate antimony anti color development-UV Spectrophotometry method; total potassium (TK) was measured by using NaOH melting-flame photometry method; available potassium (AvK) was analysized by using NH_4_OAc extraction-flame photometry method, and exchangeable calcium (Ca) was determined by using atomic absorption spectrophotometry method.

### Data analysis

Slope aspect was divided into four slope directions: (1) shaded slope (0°–45° and 315°–360°); (2) half-shaded slope (45°–90° and 270°–315°); (3) half-sunny slope (90°–135° and 225°–270°); and (4) sunny slope (135°–225°). Defined values were assigned to the qualitative variables, aspect and position. The sunny slope was assigned 0.3, the half-sunny slope was assigned 0.5, the half-shaded slope was assigned 0.8, and the shady slope was assigned 1.0. The top of the slope was assigned 0.4, the mid-slope was assigned 1.0, and the downslope was assigned 0.8.

The number of individual understory plants per plot and the Shannon–Wiener diversity (*α*-diversity) and its components (i.e., species richness (*S*), Shannon–Wiener index (*H′*), and evenness) were used to describe vegetation biodiversity. These indices were calculated with PC-ORD version 5.0^38^, using the following equations:

1 $$N=\sum {N}_{i};$$

2 $${H}^{^{\prime}}=-\sum_{i=1}^{s}ln{p}_{i}$$

3 $$E={H}^{^{\prime}}/{ln}_{s}$$

where *N*_i_ is the number of individual understory plants per plot, *S* is the species richness, and *p*_i_ is the relative abundance of species *i*, calculated as the number of plants of species *i* as a proportion of the total number of plants of all species in the *j*th plot.

Duncan's multiple comparison tests were used to test for differences in overstory and understory characteristics with soil and topographic factors among the three vegetation types. Pearson correlation analysis was used to detect relationships between environmental factors and overstory and understory flora. Differences in understory flora species composition were tested with the means of the pair-wise multi-response permutation procedure (MRPP), using a presence/absence species data matrix with rank transformed Sorensen (Bray–Curtis) distance measures. MRPP provides a *T* statistic that describes the separation between groups (the more negative *T* is, the stronger the separation is) and its associated significance^45^. An *A* value is provided to estimate the within-group similarity, with values ranging from 0 to 1, where 1 indicates identical items within the group and 0 is equal to chance expectations. The *P* value is used to indicate the significance level of the corresponding *T* statistic. Indicator species analysis (ISA) was performed to test the indicator species in each vegetation type. The indicator value of each species was assessed for significance in relation to an *α-*value (*α* = 0.05) with a Monte Carlo test based on 9,999 permutations. MRPP and ISA were performed using PC-ORD version 5.0.

Canonical correspondence analysis (CCA) was performed to examine the influence of environmental factors on understory plant distribution. We used CCA with forward-selected explanatory variables to test the effects and significance of each variable. Furthermore, variation partitioning was conducted to partition the pure effects of soil factors and topographical variables. CCA was performed with the vegan package of R 3.6.2.

## Results

### Characteristics of understory flora, soil, and topographic properties in the three vegetation types

A total of 186 understory plants, belonging to 147 genera and 76 families, were recorded. Among these plants, 91, 85, and 138 species were recorded in shrubland, forest–shrub, and mixed forest, respectively. The forest–shrub had the fewest species but the highest value for species diversity (i.e., *α*-diversity index; Table 1). The mixed forest contained the most species, while the shrubland had the lowest value for the *α*-diversity index.

Soil pH and TK were significantly higher in the mixed forest than the other two vegetations. SOM, TN, and AvN were significantly lower in the shrubland than the other two vegetations. Soil AvP and Ca were significantly higher in the mixed forest than in the shrubland, and soil AvK was significantly higher in the shrubland and the mixed forest than in the forest–shrub. However, soil TP did not significantly differ among the three vegetation types. The mixed forest showed the highest value for Rock, and the forest–shrub exhibited the highest value for Degree. Of note, the observed forest–shrub was distributed at higher elevation.

### Correlations of environmental factors with canopy characteristics and understory flora

Overstory tree density (O-density) was significantly negatively correlated with TK, AvP, AvK, and ratio of rock bareness. Exchangeable calcium was significantly negatively correlated with overstory evenness (O–E) and Shannon diversity (O–H) (Table 2). Except for SOM, TP, and exchangeable calcium, significantly positive correlations of environmental variables with overstory mean height were observed, and significantly positive correlations between overstory mean DBH and soil properties (except for SOM and TP) were also found. Overstory species richness was significantly positively related to understory species richness and Shannon diversity, and the relationships of overstory Shannon diversity with understory species richness and Shannon diversity were also significantly positive. Soil parameters, i.e., SOM, TN, AvN, and AvP, were significantly negatively correlated with individual numbers of understory (U-individual), but the correlation between understory species richness (U-*S*) and soil pH was significantly positive (Table 2). In addition, the slope aspect was significantly positively related to understory species richness (U-*S*), evenness (U-*E*), and Shannon diversity (U-*H*), but a significantly negative correlation between elevation and understory species richness (U-*S*) was found (Table 2).

**Table 2:** Correlation coefficients between environmental factors and characteristics of overstory and understory.

	O-density	O-S	O-E	O-H	O-height	O-DBH	U-individual	U-S	U-E	U-H
Soil pH	− 0.29	0.20	0.31	0.34	0.42*	0.44*	0.11	0.41*	− 0.16	0.08
Soil organic matter	− 0.20	− 0.18	− 0.15	− 0.20	0.29	0.39*	− 0.41*	− 0.30	0.04	− 0.12
Total nitrogen	− 0.26	− 0.10	− 0.08	− 0.11	0.38*	0.48**	− 0.39*	− 0.21	− 0.02	− 0.12
Total phosphorus	− 0.14	0.04	− 0.17	− 0.05	0.08	0.22	− 0.09	0.02	0.10	0.11
Total potassium	− 0.42*	− 0.16	0.06	− 0.03	0.44*	0.49**	− 0.05	0.07	0.09	0.10
Available nitrogen	− 0.32	− 0.01	0.07	0.02	0.54**	0.60**	− 0.36*	− 0.06	0.10	0.04
Available phosphorus	− 0.38*	− 0.27	− 0.09	− 0.21	0.36*	0.42*	− 0.38*	− 0.23	0.23	0.04
Available potassium	− 0.41*	− 0.11	0.03	− 0.02	0.44*	0.45**	− 0.20	0.10	0.15	0.16
Exchangeable calcium	− 0.03	− 0.33	− 0.35*	− 0.40*	0.31	0.39*	− 0.19	− 0.29	0.03	− 0.13
Ratio of rock bareness	− 0.42*	0.13	0.29	0.25	0.51**	0.57**	− 0.12	0.16	0.00	0.07
Slope degree	0.39*	− 0.11	− 0.48**	− 0.35	− 0.28	− 0.19	0.13	− 0.27	− 0.12	− 0.22
Slope aspect	− 0.12	0.24	0.33	0.34	0.31	0.26	0.08	0.38*	0.55**	0.49**
Slope position	0.20	0.04	− 0.28	− 0.11	− 0.18	− 0.09	0.25	0.21	0.09	0.19
Elevation	0.26	− 0.13	− 0.26	− 0.26	− 0.47**	− 0.45**	− 0.34	− 0.50**	0.14	− 0.15
U-individual	0.19	0.12	0.08	0.16	− 0.31	− 0.21				
U-S	− 0.03	0.54**	0.34	0.55**	0.29	0.24				
U-E	− 0.05	0.18	0.03	0.10	0.31	0.15				
U-H	0.04	0.48**	0.17	0.39*	0.32	0.18				

O-density means overstory tree density; O-S means overstory species richness; O-E means overstory evenness; O–H means overstory Shannon–Wiener index; O-height means overstory tree height; O-DBH means diameter at breast height of overstory; U-individual means number of individuals in understory; U-S means understory species richness; U-E means understory evenness; U-H means understory Shannon–Wiener index.

*P < 0.05;** *P* < 0.01.

### Effect of vegetation type on understory flora

MRPP showed that the three vegetation types were significantly different (*P* < 0.0001) from one another with respect to understory species composition, with the greatest difference being detected between the shrubland and the mixed forest (Table 3). Overall, the foreset–shrub was more similar than the shrubland in species composition to the mixed forest (Table 3).

**Table 3:** Summary of multi-response permutation procedure tests on similarity in understory species composition among contrasting forest patch types.

Forest patch types pair-wise comparison	*T* statistic^a^	*A* value^b^	*P* value^c^
Shrubland vs. forest-shrub transition	− 6.42	0.071	< 0.0001
Shrubland vs. mixed-species broadleaf forest	− 7.24	0.072	< 0.0001
Forest-shrub transition vs. mixed-species broadleaf forest	− 5.01	0.041	< 0.0001

^a^ Separation among groups by Sorensen (Brey-Curtis) distance; separation strengthens with more negative values.

^b^ Within-group similarity; the value ranges from 0 to 1, 1 indicating identical items within the group.

^c^ Significance level of the corresponding *T*-statistic.

Of the 186 understory species, ISA identified 21 species as significant indicators. The three communities had different indicator species. Thirteen species were identified as indicators of the shrubland, two of the forest–shrub, and six of the mixed forest (Table 4). Indicators of the shrubland were mainly herbaceous seed plants, while those of the mixed forest were mainly ferns.

**Table 4:** Indicator values (IV) of major species in the understory per vegetation type.

Forest patch type	Species	Value (IV)	*P* value
Shrubland	*Apluda* *mutica*	42.3	0.013
	*Barleria* *cristata*	33.3	0.015
	*Bauhinia* *corymbosa*	44.4	0.001
	*Caryopteris* *incana*	33.3	0.018
	*Chromolaena* *odorata*	63.0	0.000
	*Cratoxylum* *cochinchinense*	55.6	0.000
	*Cudrania* *tricuspidata*	44.4	0.002
	*Mallotus* *philippensis*	33.3	0.016
	*Microstegium* *nodosum*	62.3	0.000
	*Miscanthus* *floridulus*	33.3	0.015
	*Phyllanthus* *emblica*	48.3	0.003
	*Senecio* *scandens*	37.4	0.015
	*Viburnum* *propinquum*	39.9	0.036
Forest-shrub transition	*Lepionurus* *sylvestris*	50.4	0.002
	*Ulmus* *parvifolia*	47.3	0.003
Mixed-species broadleaf forest	*Asplenium* *sampsoni*	34.2	0.021
	*Asplenium* *saxicola*	33.3	0.021
	*Boehmeria* *nivea*	63.1	0.000
	*Laportea* *violacea*	51.2	0.003
	*Nephrolepis* *auriculata*	51.2	0.003
	*Streblus* *tonkinensis*	33.3	0.025

### Controls of soil and topographic factors on understory flora

**Figure 1:**
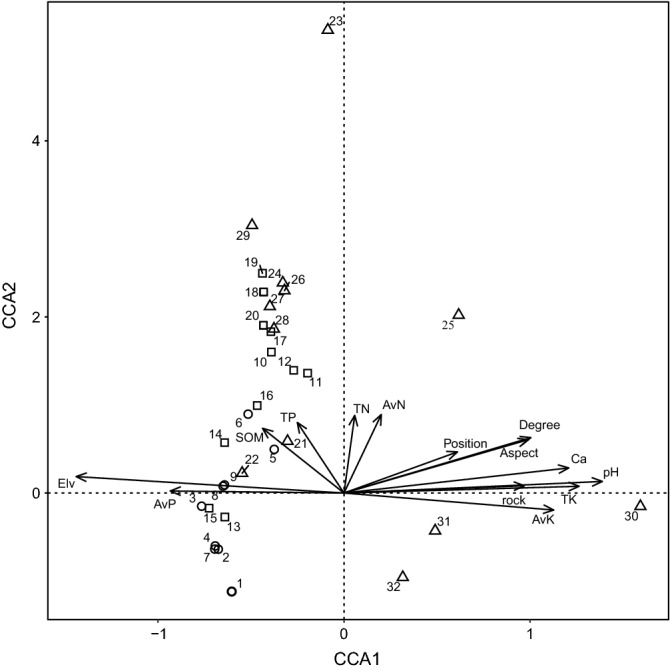
The canonical correspondence analysis (CCA) ordination of 32 plots and environmental factors. Arrows indicate the environmental variables [soil pH, soil organic matter (SOM), total nitrogen (TN), available nitrogen (AvN), total phosphorus (TP), available phosphorus (AvP), total potassium (TK), available potassium (AvK), exchangeable calcium (Ca), the ratio of rock bareness (Rock), elevation (Elv), slope degree (Degree), slope aspect (Aspect), and slope position (Position)]. Plots are identified by vegetation types as shrubland (circle), forest-shrub transition (square), and mixed forest (triangle).

For the CCA ordination, a Monte Carlo permutation test showed that the eigenvalues for the first and those for all canonical axes clearly achieved statistical significance (*P* < 0.01), indicating the soil and topography had significant comprehensive effects on the distribution of understory plants. The eigenvalues of the four axes were 0.982, 0.857, 0.764, and 0.698. The coefficients describing the species-environment correlations were 0.999, 0.981, 0.988, and 0.970. The four axes in the CCA ordination explained 50.7% of the variance in the relationship between understory flora and the environmental variables. The first axis was strongly and significantly correlated to Elv, followed by soil pH, TK, Ca, AvK, slope degree, slope aspect, rock bar, and AvP. The second axis was strongly related to AvN, followed by TN, TP, and SOM (Table 5, Fig. 1). Because the eigenvalues of the first two axes were greater than those of the others, we considered factors that were highly correlated with the first two axes to have greater effects on the understory flora.

**Table 5:** Correlation coefficients of soil and topographic factors for the first four species axes.

	Axis 1	Axis 2	Axis 3	Axis 4
Soil pH	0.933**	0.093	0.003	0.069
Soil organic matter	− 0.294	0.520**	− 0.168	0.276
Total nitrogen	0.039	0.623**	− 0.178	0.267
Total phosphorus	− 0.169	0.566**	− 0.251	0.002
Total potassium	0.849**	0.054	− 0.119	− 0.027
Available nitrogen	0.135	0.630**	0.085	0.213
Available phosphorus	− 0.628**	0.016	0.066	− 0.136
Available potassium	0.758**	− 0.136	− 0.123	− 0.013
Exchangeable calcium	0.812**	0.201	0.086	0.164
Ratio of rock bareness	0.652**	0.059	− 0.138	0.081
Slope degree	0.675**	0.445**	− 0.158	0.259
Slope aspect	0.667**	0.429*	0.354*	− 0.221
Slope position	0.410*	0.330	− 0.402*	− 0.154
Elevation	− 0.968**	0.132	− 0.073	0.094
Eigenvalues	0.982	0.857	0.764	0.698
Species-environment correlations	0.999	0.981	0.988	0.970
Cumulative percentage variance				
Of species data	7.9	14.8	21	26.6
Of species-environment relation	15.1	28.2	40	50.7

Eigenvalues and cumulative percentages of variance were explained by canonical correspondence analysis ordinations.

*P < 0.05; **P < 0.001.

Forward selection of the variables showed that Elv had the greatest explanatory power, followed by soil pH, TK, Ca, Aspect, Degree, and AvK, showing that the distribution of understory species was primarily affected by topography (Table 6).

**Table 6:** Summary of forward selection in canonical correspondence analysis.

	Explanatory power	*F*	*P*
Elevation	0.952	2.496	0.002
Soil pH	0.895	2.336	0.002
Total potassium	0.806	2.086	0.004
Exchangeable calcium	0.806	2.085	0.002
Slope aspect	0.793	2.05	0.002
Slope degree	0.785	2.028	0.004
Available potassium	0.721	1.852	0.002
Total phosphorus	0.566	1.436	0.06
Total nitrogen	0.555	1.407	0.088
Slope position	0.555	1.406	0.08
Ratio of rock bareness	0.553	1.401	0.138
Available phosphorus	0.541	1.37	0.114
Soil organic matter	0.527	1.333	0.102
Available nitrogen	0.494	1.246	0.222

The pure and overlapping effects of soil and topographic variables were shown in Table 7. The soil and topographic variables jointly explained 52.53% of the variation in understory plant distribution, of which 30.23% and 17.73% were explained by pure soil factors and pure topographic variables, respectively. Overlapping effect between soil factors and topographic variables was 4.57%. The residual fraction was 47.47%.

**Table 7:** Partition the variation of understory species abundance on soil and topographic variables.

Factors	
Species abundance variance explained by pure soil environmental factors/%	30.23
Species abundance variance explained by pure spatial factors/%	17.73
Species abundance variance explained by crossed spatial-environmental factors/%	4.57
Unexplained species abundance variation/%	47.47

## Discussion

The canopy of a plant community determines the understory flora over a large part of the area. Many studies have found that stand types have significant effects on floristic composition, species diversity, and cover of understory vegetation^29,46–48^. For instance, herbaceous species were found to cluster into different groups corresponding to contrasting woody plant canopies^49^. The three vegetation types investigated significantly differed in overstory tree composition, canopy structure, and selected soil variables except for TP (Table 1). Meanwhile, the canopy plant diversity was significantly correlated with that of the understory (Table 2). The shrubland had not yet closed for the lowest height and DBH. Light-demanding Gramineous and Compositae plants, i.e. *Apluda* *mutica*, *Microstegium* *nodosum*, *Miscanthus* *floridulus*, and *Chromolaena* *odoratum*, were common and dominant in the shrubland (Table 4). The crown density of forest–shrub transition community was larger than that of shrubland with higher height and DBH. Shade-tolerant shrub, *Lepionurus* *sylvestris*, appeared in the understory, companying with light-demanding species, *Ulmus* *parvifolia* (Table 4). The canopy of mixed forest was dense with the highest height and DBH (Table 1). Shade-tolerant ferns, i.e. *Asplenium* *sampsoni* and *Asplenium* *saxicola* etc., were common in the understory and were detected as the indicators of this community. The result reveals the contrasting canopy types may control the understory species composition by light regimes.

In this study, the species richness and *α*-diversity of the overstory usually increased from the shrubland to the forest (Table 1). Community structure became more complex and provided more diverse habitats for understory plants occupying different niches; thus, the understory species richness very markedly increased with increasing overstory species richness and *α*-diversity, with the mixed forest having the most understory species. The understory *α*-diversity also significantly increased, with the greatest *α*-diversity index being detected in the forest–shrub (Table 1, Table 2). In a karst ecosystem in China, the species diversity rose steadily with community succession, where the vine-shrub community had the most abundant species, and the highest *α*-diversity index was detected in secondary forests, and then the diversity decreased in old-growth evergreen broad-leaved forest^50^. Plant diversity generally increases with succession and tends to be exceptionally high in successional communities, while it tends to be exceptionally low in oldfield ecosystems when there is strong species dominance^51^. Our results demonstrate that plant diversity tends to increase with direct community succession from the shrubland to the forest, but the communities have not yet reached steady stages^50^. The mixed forest supported the most species in the overstory and understory; it plays a key role in maintaining plant diversity in the study area, so it is very necessary to take measures to protect the mixed forests in the degraded karst areas.

The forest canopies also support different understory flora by modifying the environment. Studies concluded that forest types controlled the understory flora by influencing soil nutrient availability^52,53^. In addition, topographies also are important factors affecting plant distribution. Several studies explored the effect of topographical variables on plant spatial patterns in the karst areas of China^54–56^, but only few focus on the coupling effect of soil and topographies on understory flora^57^. Our study detected that canopy structure influenced on understory flora through affecting on soil pH, SOM, TN, AvN, and AvP, and elevation and slope aspect were also significantly related to overstory structure and understory species diversity (Table 2). CCA revealed that elevation was the most important factor correlated with the variation in understory species distribution, followed by soil pH, TK, and Ca (Table 6), it also significantly negatively correlated with soil pH, TK, and Ca (Table 5). The shrubland was mainly distributed at higher altitude region, and light-demanding herbaceous seed plants were common in this community. Karst calcicole trees, such as *E*. *hsienmu*, *S*. *rotundifolium*, and *Lysidice* *rhodostegia*, assembled at lower altitude with soil rich Ca content to a dense mixed-species broadleaf forest. Light-demanding understory species disappeared from this community for weak light condition, but shade-tolerant calcicole, i.e. *Asplenium* *saxicola*, *Asplenium* *sampsoni* and *Streblus* *tonkinensis* assembled under the canopy. Our results reveals that elevation controls the spatial distribution of vegetation and soil factors, and then the understory plants, and soil Ca content is another important factor affecting the distribution of understory plants. The soil Ca content of karst ecosystem is higher than that of non-calcareous area based materially on carbonate rock. The adaptation of calcicole to high Ca content limestone soil needs to be studied to detect the formation of plant habitat diversity. However, CCA ordination can only explain 50.7% of the variance in the understory flora, indicating that other factors also affect the understory flora and need to be further studied.

## Conclusion

Overall, the three vegetation types were significantly different (*P* < 0.0001) in understory species composition. Twenty-one understory species were identified as indicator species. Indicators of the shrub were mainly light-demanding herbaceous seed plants, while those of the mixed forest were mainly shade-tolerant calcicoles. Vegetation types controlled understory species composition by modifying understory environments. Elevation controlled the spatial distribution of vegetation and soil properties, and then the understory plants. Meanwhile, soil Ca content was also important for the understory species distribution. In view of the importance of understory for soil, water and biodiversity conservation in karst region, and the investigated communities have not yet reached a steady state and easily degenerate, it is critical to protect the mixed forest and take measures to promote the vegetation restoration in the study area.

## Data Availability

The datasets generated and/or analysed in the current study are available from the corresponding author upon reasonable request.
